# Benefits of Bandwidth Feedback in Learning a Complex Gymnastic Skill

**DOI:** 10.2478/hukin-2013-0039

**Published:** 2013-07-05

**Authors:** Jerzy Sadowski, Andrzej Mastalerz, Tomasz Niznikowski

**Affiliations:** 1 Jozef Pilsudski University of Physical Education, Warsaw, Faculty of Physical Education and Sport, Biala Podlaska, Poland.; 2 Jozef Pilsudski University of Physical Education, Warsaw, Faculty of Physical Education, Poland.

**Keywords:** guidance hypothesis, complex motor skill, knowledge of performance, gymnastics

## Abstract

The aim of this study was to examine the effects of two different frequencies of feedback during the process of learning a complex gymnastic skill, the round-off salto backward tucked. Thirty male acrobats participated in the study. They were randomly assigned to two groups: B - bandwidth feedback (n=15) or C - 100% feedback (n=15). Group B was provided with error information regarding the key elements of movement techniques only (bandwidth feedback). Our research demonstrates the advantage of augmented feedback information related to errors in the key elements. Information about errors in the key elements during learning a complex gymnastic skill prevents the gymnast from becoming overwhelmed, which promotes better motor control. These results provide support for the generalisation of bandwidth feedback principles to a complex task. Our research shows that the guidance hypothesis can also be tested in practical settings for a complex movement task.

## Introduction

One of the most important factors in the process of learning a motor skill is the feedback provided to the learner. Many researchers have attempted to identify the most appropriate method of providing information through feedback for a person who is learning or refining motor skills (for reviews, see Adams, 1987 ; Newell, 1976 ; Salmoni et al., 1984 ; Swinnen, 1996 ; Wulf and Shea, 2004 ; Sadowski et al., 2009 ). The bulk of the existing research provides strong experimental evidence for factors such as the frequency of feedback, organisation of feedback, types of augmented feedback, forms of knowledge of result (KR) and knowledge of performance (KP) (for reviews, see Kluger and De Nisi, 1996 ; Schmidt and Lee, 1999 ; Winstein and Schmidt, 1990 ; Sadowski et al., 2011 ). Researchers have found evidence that too frequent feedback has a degrading effect on delayed retention and transfer tests. This phenomenon is called the guidance hypothesis (for reviews, see Salmoni et al., 1984 ; Swinnen, 1996 ; Wulf and Shea, 2004 ). According to the guidance hypothesis, augmented information may have positive effects guiding the learner to the correct response or negative effects on motor-skill learning if it is provided too frequently or if it is in a form that is too easy to use ( Salmoni et al., 1984 ; Schmidt and Lee, 1999 ). Researchers have proposed some explanations for the negative effects of guidance on motor learning ( Schmidt, 1991 ; Young and Schmidt, 1992 ). From an information-processing perspective, one hypothesis is that a learner becomes overly dependent on augmented feedback during acquisition and may not process information from the intrinsic sources of the task, which is critical for performing the task without KR ( Salmoni et al., 1984 ; Schmidt, 1991 ). Another explanation for the negative effect of guidance is that frequent KR encourages the learner to make too many corrections during practice (i.e., maladaptive, short-term corrections), leading to an inability to recognise and produce stable behaviour in retention ( Schmidt, 1991 ). This hypothesis has been tested mainly in experiments on KR involving a simple motor task.

As many studies have revealed, principles derived from the study of learning simple skills are not necessarily generalisable to the process of learning complex skills (for reviews, see Wulf and Shea, 2002 ). Therefore, to understand the processes of learning complex motor skills and to provide recommendations for teaching these skills, it is important to examine the process of learning complex skills. Researchers who have studied the effects of reducing the frequency of KR during the acquisition of complex motor skills have identified counterproductive results ( Maslovat et al., 2009 ; Butki and Hoffman, 2003 ; Smith et al., 1997 ; Wulf et al., 1998 ). Several researchers have observed that reduced feedback frequency is of little benefit in the process of learning complex skills ( Kohl and Guadagnoli, 1996 ; Lai and Shea, 1999 ).

Wulf et al. (1998) demonstrated that frequent feedback enhanced the process of learning a ski-simulator task. Similarly, Swinnen et al. (1997) showed the beneficial effects of frequent feedback on learning a two-handed coordination task. According to Baudry et al. (2006) , concurrent feedback benefits the circle movement performed on a pommel horse.

One of the methods of providing KR or KP is known as bandwidth knowledge of results (BKR). With this method, the nature of the augmented feedback is based on a specified bandwidth or an acceptable range of error. Bandwidth feedback is a technique that reduces the relative frequency of feedback ( Magill, 1993 ; Lee and Maraj, 1994 ; Butler et al., 1996 ; Smith et al., 1997 ).

It is well documented that bandwidth feedback is highly effective for learning simple motor skills during physical practice ( Butler et al., 1996 ; Cauraugh et al., 1993 ; Lee and Carnahan 1990 ; Park et al., 2000 ; Sherwood, 1988 ; Smith et al., 1997 ) and observational practice ( Badets and Blandin, 2005 ; Badets and Blandin, 2010 ). However, experimental evidence of the effectiveness of applying bandwidth feedback and supporting the guidance hypothesis in learning complex motor skills is somewhat ambiguous ( Baudry et al., 2006 ; Lai et al., 2000 ; Maslovat et al., 2009 ; Smith et al., 1997 ; Swinnen et al., 1997 ; Wulf et al., 1998 ). Hence, it is not clear whether the different types of instruction benefit or hinder the learning and performance of complex motor skills, especially in practical settings. Feedback that is too precise is as useless as feedback that is too vague ( Wright et al., 1997 ). Lee and Carnahan (1990) have postulated that the amount and precision of KR are often overwhelming, exceeding the learner’s ability to correct the response because learners can effectively process only a limited amount of information at a time. In practical settings, teachers or coaches typically provide learners with demonstration and verbal feedback. To enforce the efficacy of motor learning, demonstration should be accompanied by sufficient verbal instruction aimed at ensuring that the learner’s attention is focused on the key elements of a task ( Masser, 1993 ; Mawer, 1990 ; Magill, 1998 ). Hence, the teacher should identify the key elements to avoid overwhelming the learner’s long-term memory.

In the process of learning a complex gymnastic skill, it is assumed that information about errors in key elements is more effective than feedback provided after each error. Therefore, the aim of this study was to examine the effect of two different frequencies of feedback during the process of learning a complex gymnastic skill, a round-off salto backward tucked.

## Methods

### Participants

Thirty male acrobats participated in the study. These acrobats were 11±0.3 years of age, and all had between one and two years of experience in acrobatics competitions at a regional level in Poland. Prior to participating in the study, all of the acrobats were fully informed about the protocol, and their parents provided written consent. The acrobats were randomly assigned to one of two groups: bandwidth feedback (B, n=15) or 100% feedback (C, n=15). The participants performed a correct execution (in the range of 9.0 to 9.2 points, in accordance with the code of points [ FIG, 2001 ]) of the basic gymnastic routines: handstand, cartwheel, and somersault. Differences in the execution of gymnastic routines between groups B and C were insignificant. The study was approved by the Ethic Committee of Human Experiments at Warsaw University of Physical Education.

### Procedure

All participants completed four practice sessions per week on a Monday-Tuesday-Thursday-Friday schedule for sixteen weeks. The progressive-part method was employed in this study. The whole gymnastic skill (round-off salto backward tucked) was broken down into components that characterised the full movement: initial position, flight phase and final position. Each component and the whole task were learned separately during sixteen subsequent training sessions. Prior to practicing, all of the participants received a demonstration of the task along with verbal guidance. The demonstration was given by an expert coach. Subsequent to the demonstration, the participants were asked to perform at their best each time. Afterwards, all of the participants were provided with prescriptive verbal feedback about the errors they made and also about how to correct them (specific for each individual). The participants in group B received feedback only for those trials in which they executed the task with errors in key elements; other errors were neglected within an acceptable range of errors. The participants in group C received feedback about all of the errors they made during each trial (100% feedback), which is a typical routine in gymnastic training settings. The quantity of guidance was recorded at each training session. Information about errors in group B was related only to the key elements of movement techniques, and the guidance was only approximately ***x̄*** = 5.5±1.2 of the average information ( ***x̄*** =7.9±1.1) applied in group C at one training session. Information about performance in group C referred to each committed error (100% frequency). In both groups, lack of information about errors had to be treated as an exercise that was executed correctly. Both groups performed ten trials during each training session for a total of 640 trials. Between trials, there were 120 s breaks. The pre-test (one day before the experiment), retention test (one day after the experiment) and delayed retention test (one week after the experiment) consisted of three trials of the roundoff salto backward tucked.

### Estimation of key elements

**Figure 1:**
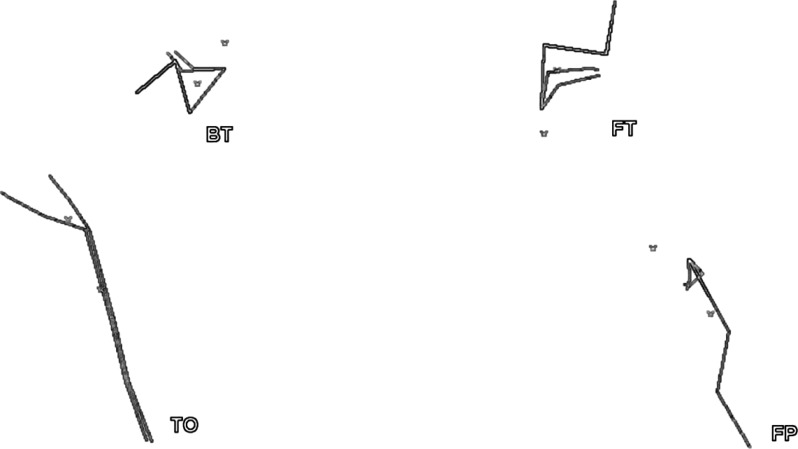
*
The body positions following the key elements that were subjected to analysis: TO - initial body position, BT - body position at the beginning of tucking, FT - body position at the end of tucking and FP - final body position.
*

All of the information characterised the key elements’ validity and their impact on the performance of a round-off salto backward tucked based on information received from coaches. Twenty-six coaches with practical experience ( ***x̄*** =23.4±5.1 ) marked the key elements in the round-off salto backward tucked when it was visualised on a computer monitor after being recorded at 0.02 sec intervals. Only the body position with a coefficient of convergence higher than 0.9 was considered to be a key element. The following key elements were subjected to analysis ( Figure 1 ): initial body position (biomechanically expedient position of the acrobat’s body on support in the system of coordinates creating effective conditions for the takeoff - TO), body position at the beginning of tucking (during the ascending phase of the flight - BT), body position at the end of tucking (during the descending phase of the flight - FT) and the final body position (touchdown - FP).

### Estimation of experimental effects based on performance quality

The performance of the acrobats was used to evaluate the proximity of the key elements to the expert model. Model values were calculated based on video analysis of elite acrobats (n=7) aged 18.4±1.2 who were members of the Polish national team. The values were subsequently applied to define the rate of technical compliance (RC). The RC value was defined as the relative difference of the angle in each joint between elite acrobats and both training groups. The RC was calculated twice for each joint during the pre-test and retention measurements. The best performance was selected to obtain the RC. All of the best trials were recorded with two NTSC (60 Hz) video cameras and APAS 2000 (Ariel Dynamics) cinematographic analysis systems. Ten light-reflective markers were placed on different parts of the right side of the participants’ bodies, including the foot, ankle, knee, hip, wrist, elbow, shoulder, hand, and the centre of the head. Cameras were placed 6 m apart, 9 m from the front of the data acquisition region and at a height of 1.75 m. Motion sequences were auto-digitised, transformed, and smoothed using a low-pass digital filter (10 Hz). A digital filter of 10 Hz was used to minimise any smoothing effect on the raw data and to avoid masking any inherent system error. The accuracy of the three-dimensional linear and angular values was estimated based on the procedure described by Klein and DeHaven (1995) . A composite control cube consisting of 22 reflective calibration points and 10 data points that were placed on the acrobats’ bodies was digitised and entered into the three-dimensional linear transformation (DLT) module and converted to real displacements. The average error of the marker position determined for all measurements was 2.88 mm (1.2%) with the participant 9 m away from the camera.

### Estimation of experimental effects based on judges’ scores

Three qualified judges viewed each trial and provided a performance score. Breaks in body segmental alignment during each test were penalised by a deduction of 0.10 to 0.50 points (on a scale of 10 points), in accordance with the code of points ( FIG, 2001 ). The average of the three professional judges’ assessments was the final score of the performance.

### Statistical analysis

Analysis of variance for repeated measures (ANOVA) was used to estimate the statistical significance of differences among measurements. The normality of distribution and the homogeneity of variances were tested with the Shapiro-Wilk test. After the verification of the prerequisite, studied variables were analysed using a two-way mixed-factor analysis of variance, Group (2) × Test Time (3), with the two experimental groups of acrobats representing a between- participants factor and the testing times (pre-test, retention and delayed retention tests) representing a within-participants factor. A probability level of p<0.05 was used to indicate statistical significance. For significant differences, a Fisher post hoc test was used. The results were statistically analysed using the Statistica programme (StatSoft, Inc. [2005]. STATISTICA [data analysis software system], version 7.1. www.statsoft.com).

## Results

**Figure 2:**
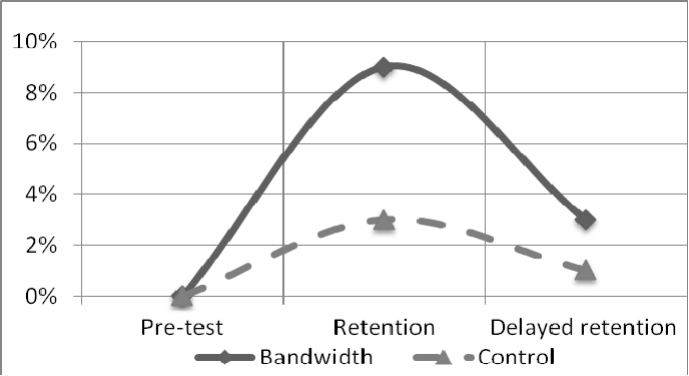
*
Mean scores for the technical performance of the round-off salto backward tucked.
*

To assess the performance quality, the judges’ scores were used (a typical evaluation method in competitions). Scores for performing the round-off salto backward tucked were converted into percentage values. It was assumed that 100% equalled 10 points, which was the maximum score for performing a given motor task ( Figure 2 ), and acrobats’ errors caused percentage values to be deducted from the total. At the beginning of the experiment (pre-test), the differences between the key elements and the mean values obtained by groups B and C for performing the round-off salto backward tucked were not significant (a group effect F(1, 28)= 0.33, p=0.57; d=0.22)). In the pre-test, group B made more errors while performing the round-off salto backward tucked than group C (statistically insignificant).

The experiment effect was analysed using ANOVA with repeated measures (Group × Test Time). ANOVA revealed a significant effect of Group (F(1,28)=54,87, p<0.001) as well as a significant Test Time effect (F(2, 56)=190,74, p<0.001). The post hoc comparison indicated significant differences between the feedback applied in both groups during the retention test (6%; p<.001; size effect d=3.18) and delayed retention test (7%; p<0.001; size effect d=3.65).

**Figure 3:**
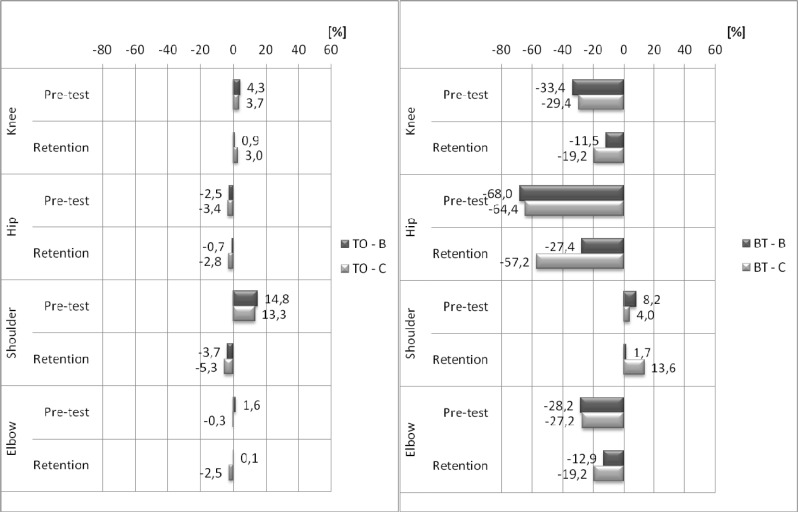
*
Means of the rate of technical compliance (RC) during pre-test and retention.
* *
B – bandwidth group, C – 100% feedback group. TO - initial body position, BT - body position at the beginning of tucking, FT - body position at the end of tucking and FP - final body position.
*

**Figure 4:**
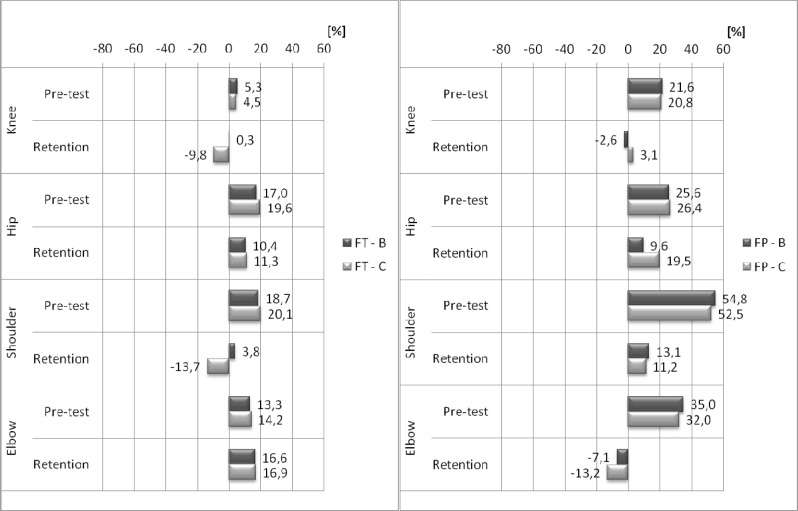
*
Means of the rate of technical compliance (RC) during pre-test and retention.
* *
B – bandwidth group, C – 100% feedback group. FT - body position at the end of tucking and FP - final body position.
*

According to the judges’ scores, the type of feedback has a significant influence on performance between the retention and the delayed retention tests. The scores improved significantly in group B (3%; p<0.001; d=2.37) and insignificantly in group C (0.4%; p=0.187; d=0.42). The impact of the feedback on the correctness of the task performance was also assessed by RC in four key elements: TO, BT, FT and FP ( Figure 3 and 4 ).

The results revealed that joint angles at TO were not significantly different between groups B and C during the pre-test and retention test. All groups improved the retention tests’ RC to the model’s pattern at BT, FT and FP (except the shoulder in BT for group C and the elbow in FT). However, this effect was greater for group B. The advantage of group B was most pronounced at BT. ANOVA revealed a significant group main effect during the retention test: the knee joint (7.7%, F(1, 28)= 4.25, p=0.049; d=0.78), the hip joint (29.7%, F(1, 28)= 6.85, p=0.014; d=0.99), the shoulder joint (12.00%, F(1, 28)= 5.63, p=0.025; d=0.89) and the elbow joint (6.4%, F(1, 28)= 5.13, p=0.031; d=0.86). FT was the only element in which joint angles insignificantly deteriorated in the elbow joint in both groups as well as in the knee joint in group C. Furthermore, a significant group main effect concerning angle values of the knee joint and the shoulder joint were found in the retention test (10.1%, F(1, 28)=4.99, p=0.033; d=0.84 and 17.4%, F(1, 28)=5.84, p=0.022; d=0.91, respectively). A significant improvement of RC to the model’s pattern was also observed in the FP element. However, the differences between the groups in terms of joint angles did not exceed 6% during the retention test, and there was no significant difference between the groups in the pre-test. These results show that both groups improved their RC to the model pattern. The group main effect was revealed only for the hip joint during the retention test (10%, F(1, 28) 4.33, p=0.047; d=0.79).

## Discussion

The aim of this study was to examine the effects of two different frequencies of feedback during the process of learning a complex gymnastic skill, a round-off salto backward tucked. By manipulating the amount of verbal prescriptive feedback (bandwidth feedback vs. 100% frequency feedback), we determined the benefit derived from providing bandwidth feedback in learning a complex gymnastic skill. The results indicated that the quantity of errors in KP and the methods of their correction may differentiate the effects of learning complex exercises with many degrees of freedom of the body. The performance of the round-off salto backward tucked consists of integrating several motor actions with the objective of reaching an identified goal. Therefore, in these studies, the correctness of the performance was assessed using RC and the judges’ scores. A pronounced increase in the scores of both groups was observed ( Figure 2 ). Group B outperformed group C. These differences may have been mediated by different KP frequencies. The frequency of information about errors was significantly lower in group B; however, in both groups, the frequency of feedback was reduced during the experiment as a whole. The differences in the judges’ scores for the round-off salto backward tucked were even greater after the feedback was removed.

Although the judges’ ratings must be applied to the task as a whole, the differences in specific angular positions recognised as models by gymnasts must be described by the RC ratio. Both scales should provide similar conclusions. It is essential to highlight the effects of the feedback information applied in group B on the knee joint angle in the initial body position (take-off phase – TO), the flight (descent – FT) and the final body position (landing – FP). In three out of four key elements, the values were almost identical to the model. Compared with the model, the hip joint angle in TO had a wider range of motion in group B and a narrower range of motion in group C. For the other key elements, it was not possible to obtain values similar to the model values. Nonetheless, the trend observed in Figure 3 and 4 characterised the positive effects of the feedback applied in both groups. Statistically significant angle changes in the hip joint were attained by group B at the beginning of tucking (BT) and FP. The angle values of the shoulder joint in the FP position changed significantly (p<0.01). Body position improved in the other key elements after feedback information was applied to group B. Thus, the participants improved their proximity to the model. With respect to FT, the errors were not completely eliminated in either group. From the beginning, both groups of acrobats demonstrated correct joint angles in TO. Both methods contributed to an increase in RC in FP (landing).

These results partially confirm the effects observed by researchers of the guidance theory. In practical settings, feedback that was applied to both of the groups in our research produced a decrease in the number of errors and an increase in the accuracy of the key elements and the execution of the task as a whole. However, the lower frequency of feedback in group B was more effective in comparison to that of group C in the retention and delayed retention tests. Our data do not strictly agree with the findings of many authors who claim that more frequent feedback is more beneficial than less frequent feedback for learning complex motor skills ( Wulf et al., 1998 ; Swinnen at al., 1997 ; Baudry et al., 2006 ). Information about the key elements during acquisition was sufficient to achieve a significant improvement in task quality. Our findings corroborate those of Smith et al. (1997) , who found that participants practicing a golf chipping task who received information with wider bandwidth criteria performed the task more consistently in retention than the group under low bandwidth conditions (0 to 5%). These results provide support for the generalisation of bandwidth feedback principles to a complex task. The amount of feedback information influenced the effects obtained during retention differently. In both scoring systems, the results improved significantly in group B and insignificantly in group C. A similar effect was observed by Tzetzis and Votsis (2006) for the group that received only instructional cues on errors in the execution of the forehand long serve. Furthermore, Butki and Hoffman (2003) showed that a group performing a continuous golf putting task performed better during acquisition, but the KR-deprived groups performed better in retention trials. In other studies, Maslovat et al. (2009) contrasted two types of concurrent augmented feedback that differed in the amount of information offered during the acquisition process. The results showed that the group receiving continuous feedback outperformed the discrete feedback group throughout the acquisition period. However, during retention, the discrete feedback group showed an increase in performance, whereas the continuous feedback group showed deterioration. These results are similar, in part, to the results obtained in our study. Our results support the guidance theory because the participants who received a large amount of feedback in the process of learning a complex motor skill (acquisition phase) became reliant on the feedback more than the group that received less external information. Results obtained by Maslovat et al. (2009) showed that feedback communicated at discrete points resulted in an increase in performance during the retention test, which is similar to the situation in which the bandwidth group receives information only about errors in key elements. However, Baudry et al. (2006) found that auditory concurrent feedback benefited the circle movement performed on a pommel horse during acquisition, and the results of a retention test did not show a decline in performance. It is worth noting that the participants received information about key elements (the upper back and the popliteal area of the right knee). These results may have occurred because the participants were able to process the information without becoming overwhelmed and were therefore able to use an internal source of information more effectively. Kernodle et al. (2001) conducted an experiment on learning an overarm throw that examined the effectiveness of verbal instructions for correcting errors versus verbal instructions in addition to watching a videotape replay. The only significant difference was that the rated throwing form for retention by the group receiving verbal instruction alone had a higher mean rating than that of the group that was given the combined information. The videotape replay may not have enhanced learning because too much information may have interfered with learning. Magill and Schoenfelder-Zoholi (1996) supported this supposition by stating that the use of a model in addition to prescriptive KP may provide redundant information for novices learning a new skill.

Excessive detail in verbal cues can overload the learner and disrupt motor activity ( Hicks, 1975 ). Some researchers have combined the effect of feedback with the complexity or the type of skill content and the learning stage of the participants ( Magill, 1998 ; Kernodle and Carlton, 1992 ; Tzetzis and Votsis, 2006 ). According to Gentile (1987) , “What takes place during learning is very much task dependent”. Researchers do not agree on the type of feedback that is most suitable for learning various skills ( Laguna, 2008 ). One of the basic concepts of effective feedback is that the content of the information provided should match the aspects that the performer can control ( Schmidt, 1991 ) and must control ( Whiting and Vereijken, 1993 ). Feedback should help to change the movement, but this information is useful only if the acrobat is able to control the movement features for which the information is provided. The effectiveness of this type of feedback is even greater if the feedback refers to the key elements of the movement technique. Liu (2001) asserted that different opportunities for feedback produce different effects depending on the specified task goal. Our results prove that the amount of information is not the most important aspect of learning complex motor skills. Too much information appeared to be less effective for learning a gymnastics skill than task-related information given to a learner about errors in key elements of the task. Limiting the number of cues to the most critical ones, which are designed to trigger the movement sequence, may produce better results. Masser (1993) demonstrated that one cue can be successful in prompting an entire movement. In that study, critical cues helped first-grade students achieve handstands and forward rolls because the learners were cognitively aware of the important biomechanical action on which they needed to concentrate during practice.

The results in this study on the use of bandwidth feedback may be significant for instructors and coaches. Teachers and coaches must understand what information they should provide to facilitate the process of learning complex skills, how often that feedback should be provided and how precise the information should be. It is not appropriate to generalise beyond the scope of this research because this study is limited by the feedback models used for semi-experienced participants in gymnastics. Further research is needed to study the role of bandwidth feedback directed at key elements in learning complex skills with multiple degrees of freedom among elite athletes in practical settings. It remains to be investigated to what extent the principles for learning a specific complex motor skill can be generalised to learning other complex motor skills.

## References

[b1-jhk-37-183] 
Adams
 
JA
 (
1987
). Historical review and appraisal of research on the learning, retention, and transfer of human motor skills. Psychological Bulletin.

[b2-jhk-37-183] 
Badets
 
A
, 
Blandin
 
Y
 (
2005
). Observational Learning: Effects of Bandwidth Knowledge of Results. Journal of Motor Behavior.

[b3-jhk-37-183] 
Badets
 
A
, 
Blandin
 
Y
 (
2010
). Feedback schedules for motor-skill learning: the similarities and differences between physical and observational practice. Journal of Motor Behavior.

[b4-jhk-37-183] 
Baudry
 
L
, 
Leroy
 
D
, 
Thouvarecq
 
R
, 
Choller
 
D
 (
2006
). Auditory concurrent feedback benefits on the circle performed in gymnastics. Journal of Sports Sciences.

[b5-jhk-37-183] 
Butki
 
BD
, 
Hoffman
 
SJ
 (
2003
). Effects of reducing frequency of intrinsic knowledge of results on the learning of a motor skill. Perceptual and Motor Skills.

[b6-jhk-37-183] 
Butler
 
MS
, 
Reeve
 
TG
, 
Fischman
 
MG
 (
1996
). Effects of the instructional set in the bandwidth feedback paradigm on motor skill acquisition. Research Quarterly for Exercise and Sport.

[b7-jhk-37-183] 
Cauraugh
 
JH
, 
Chen
 
D
, 
Radlo
 
SJ
 (
1993
). Effects of traditional and reversed bandwidth knowledge of results on motor learning. Research Quarterly for Exercise and Sport.

[b8-jhk-37-183] FIG (2001). Code of points: Artistic gymnastics for men.

[b9-jhk-37-183] 
Gentile
 
AM
, 
Carr
 
JH
, 
Shephard
 
RB
 (
1987
). Skill acquisition: Actions, movements and neuromotor processes. Movement science: Foundations for physical therapy in rehabilitation.

[b10-jhk-37-183] 
Hicks
 
RE
 (
1975
). Intrahemispheric response competition between vocal and unimanual performance in normal adult human males. Journal of Comparative and Physiological Psychology.

[b11-jhk-37-183] 
Kernodle
 
MW
, 
Carlton
 
LG
 (
1992
). Information feedback and the learning of multiple-degree-of-freedom activities. Journal of Motor Behaviour.

[b12-jhk-37-183] 
Kernodle
 
MW
, 
Johnson
 
R
, 
Arnold
 
DR
 (
2001
). Verbal instruction for correcting errors versus such instructions plus videotape replay on learning the overhand throw. Perceptual and Motor Skills.

[b13-jhk-37-183] 
Klein
 
PJ
, 
DeHaven
 
JJ
 (
1995
). Accuracy of three-dimensional linear and angular estimates obtained with the ariel performance analysis system. Archives of Physical Medicine and Rehabilitation.

[b14-jhk-37-183] 
Kluger
 
AN
, 
DeNisi
 
A
 (
1996
). The effects of feedback interventions on performance: A historical review, a meta-analysis, and a preliminary feedback intervention theory. Psychological Bulletin.

[b15-jhk-37-183] 
Kohl
 
RM
, 
Guadagnoli
 
MA
 (
1996
). The scheduling of knowledge of results. Journal of Motor Behavior,.

[b16-jhk-37-183] 
Laguna
 
P
 (
2008
). Task complexity and sources of task-related information during the observational learning process. Journal of Sports Science.

[b17-jhk-37-183] 
Lai
 
Q
, 
Shea
 
CH
 (
1999
). Bandwidth knowledge of results enhances generalized motor program learning. Research Quarterly for Exercise and Sport.

[b18-jhk-37-183] 
Lai
 
Q
, 
Shea
 
CH
, 
Wulf
 
G
, 
Wright
 
DL
 (
2000
). Optimizing generalized motor program and parameter learning. Research Quarterly for Exercise and Sport.

[b19-jhk-37-183] 
Lee
 
TD
, 
Carnahan
 
H
 (
1990
). When to provide knowledge of results during motor learning: Scheduling effects. Human Performance.

[b20-jhk-37-183] 
Lee
 
TD
, 
Maraj
 
BK
 (
1994
). Effects of bandwidth goals and bandwidth knowledge of results on motor learning. Research Quarterly for Exercises and Sport.

[b21-jhk-37-183] 
Liu
 
UD
 (
2001
). An experimental study on the relationship between opportunity of feedback and effects of feedback in physical education instructions. Journal of Tianjin Institute of Physical Education.

[b22-jhk-37-183] 
Magill
 
RA
 (1993). Motor Learning: Concepts and Applications.

[b23-jhk-37-183] 
Magill
 
RA
 (
1998
). Knowledge is more than we can talk about: Implicit learning in motor skill acquisition. Research Quarterly for Exercise and Sport.

[b24-jhk-37-183] 
Magill
 
RA
, 
Schoenfelder-Zoholi
 
B
 (
1996
). A visual model and knowledge of performances sources of information for learning a rhythmic gymnastics skill. International Journal of Sport Psychology.

[b25-jhk-37-183] 
Maslovat
 
D
, 
Brunke
 
KM
, 
Chua
 
R
, 
Franks
 
IM
 (
2009
). Feedback effects on learning a novel bimanual coordination pattern: support for the guidance hypothesis. Journal of Motor Behaviour.

[b26-jhk-37-183] 
Masser
 
LS
 (
1993
). Critical cues help first grade students’ achievement in handstand and forward rolls. Journal of Teaching in Physical Education.

[b27-jhk-37-183] 
Mawer
 
M
 (
1990
). It’s not what you do, it’s the way that you do it! Teaching skills in physical education. British Journal of Physical Education.

[b28-jhk-37-183] 
Newell
 
KM
 (
1976
). Knowledge of result and motor learning. Exercise and Sport Science Reviews.

[b29-jhk-37-183] 
Park
 
JH
, 
Shea
 
CH
, 
Wright
 
DL
 (
2000
). Reduced-frequency concurrent and terminal feedback: A test of the guidance hypothesis. Journal of Motor Behavior.

[b30-jhk-37-183] 
Sadowski
 
J
, 
Mastalerz
 
A
, 
Niźnikowski
 
T
, 
Wiśniowski
 
W
, 
Biegajło
 
M
, 
Kulik
 
M
 (
2011
). The effects of different types of verbal feedback on learning a complex movement task studies. Pol J Sport Tourism.

[b31-jhk-37-183] 
Sadowski
 
J
, 
Niźnikowska
 
E
, 
Niźnikowski
 
T
 (
2009
). Effectiveness of teaching the basic acrobatic exercises and patterns in the case of acrobats having different coordination potential. Pol J Sport Tourism.

[b32-jhk-37-183] 
Salmoni
 
AW
, 
Schmidt
 
RA
, 
Walter
 
CB
 (
1984
). Knowledge of results and motor learning: A review and critical reappraisal. Psychological Bulletin.

[b33-jhk-37-183] 
Schmidt
 
RA
 (
1991
). Motor learning and performance: From principles to practice.

[b34-jhk-37-183] 
Schmidt
 
RA
, 
Lee
 
TD
 (
1999
). Motor control and learning. A behavioral emphasis.

[b35-jhk-37-183] 
Sherwood
 
DE
 (
1988
). Effect of bandwidth knowledge of results on movement consistency. Perceptual and Motor Skills.

[b36-jhk-37-183] 
Smith
 
PJ
, 
Taylor
 
SJ
, 
Withers
 
K
 (
1997
). Applying bandwidth feedback scheduling to a golf shot. Research Quarterly for Exercise and Sport.

[b37-jhk-37-183] 
Swinnen
 
SP
, 
Zelaznik
 
HN
 (
1996
). Information feedback for motor skill learning: A review. Advances in motor learning and control.

[b38-jhk-37-183] 
Swinnen
 
SP
, 
Lee
 
TD
, 
Verschueren
 
S
, 
Serrien
 
DJ
, 
Bogaerds
 
H
 (
1997
). Interlimb coordination: Learning and transfer under different feedback conditions. Human Movement Science.

[b39-jhk-37-183] 
Tzetzis
 
G
, 
Votsis
 
E
 (
2006
). The effect of different feedback methods on badminton skills acquisition and retention. Perceptual and Motor Skills.

[b40-jhk-37-183] 
Whiting
 
HTA
, 
Vereijken
 
B
 (
1993
). The acquisition of coordination in skill learning. International Journal of Sport Psychology.

[b41-jhk-37-183] 
Winstein
 
CJ
, 
Schmidt
 
RA
 (
1990
). Reduced feedback of knowledge of results enhances motor skill learning. Journal of Experimental Psychology: Learning, Memory, and Cognition.

[b42-jhk-37-183] 
Wright
 
DL
, 
Munyon-Smith
 
V
, 
Sidaway
 
B
 (
1997
). How close is too close for precise knowledge of results?. Research Quarterly for Exercise and Sport.

[b43-jhk-37-183] 
Wulf
 
G
, 
Shea
 
CH
 (
2002
). Principles derived from study of simple skills do not generalize to complex skill learning. Psychonomic Bulletin and Review.

[b44-jhk-37-183] 
Wulf
 
G
, 
Shea
 
CH
, 
Williams
 
AM
, 
Hodges
 
NJ
 (
2004
). Understanding the role of augmented feedback: The good, the bad, and the ugly. Skill acquisition in sport.

[b45-jhk-37-183] 
Wulf
 
G
, 
Shea
 
CH
, 
Matschiner
 
S
 (
1998
). Frequent feedback enhances complex motor skill learning. Journal of Motor Behavior.

[b46-jhk-37-183] 
Young
 
DE
, 
Schmidt
 
RA
, 
Stelmach
 
GE
, 
Requin
 
J
 (
1992
). Augmented feedback for enhanced skill acquisition. Tutorials in motor behavior II.

